# Resting-State EEG Oscillations in Amyotrophic Lateral Sclerosis (ALS): Toward Mechanistic Insights and Clinical Markers

**DOI:** 10.3390/jcm14020545

**Published:** 2025-01-16

**Authors:** James Chmiel, Marta Stępień-Słodkowska

**Affiliations:** 1 Faculty of Physical Culture and Health, Institute of Physical Culture Sciences, University of Szczecin, Al. Piastów 40B blok 6, 71-065 Szczecin, Poland; 2 Doctoral School, University of Szczecin, Mickiewicza 16, 70-384 Szczecin, Poland

**Keywords:** amyotrophic lateral sclerosis, ALS, EEG, electroencephalogram, electroencephalography, QEEG, oscillations, neurophysiology, electrophysiology, neuroimaging, neural correlates

## Abstract

**Introduction:** Amyotrophic lateral sclerosis (ALS) is a complex, progressive neurodegenerative disorder characterized by the degeneration of motor neurons in the brain, brainstem, and spinal cord. Several neuroimaging techniques can help reveal the pathophysiology of ALS. One of these is the electroencephalogram (EEG), a noninvasive and relatively inexpensive tool for examining electrical activity of the brain with excellent temporal precision. **Methods:** This mechanistic review examines the pattern of resting-state EEG activity. With a focus on publications published between January 1995 and October 2024, we carried out a comprehensive search in October 2024 across a number of databases, including PubMed/Medline, Research Gate, Google Scholar, and Cochrane. **Results:** The literature search yielded 17 studies included in this review. The studies varied significantly in their methodology and patient characteristics. Despite this, a common biomarker typical of ALS was found—reduced alpha power. Regarding other oscillations, the findings are less consistent and sometimes contradictory. As this is a mechanistic review, three possible explanations for this biomarker are provided. The main and most important one is increased cortical excitability. In addition, due to the limitations of the studies, recommendations for future research on this topic are outlined to enable a further and better understanding of EEG patterns in ALS. **Conclusions:** Most studies included in this review showed alpha power deficits in ALS patients, reflecting pathological hyperexcitability of the cerebral cortex. Future studies should address the methodological limitations identified in this review, including small sample sizes, inconsistent frequency-band definitions, and insufficient functional outcome measures, to solidify and extend current findings.

## 1. Introduction

Amyotrophic lateral sclerosis (ALS) is a complex, progressive neurodegenerative disorder characterized by the degeneration of motor neurons in the brain, brainstem, and spinal cord [1]. This leads to a loss of voluntary muscle control, resulting in muscle weakness, atrophy, paralysis, and, ultimately, respiratory failure—the leading cause of death in most patients within 3 to 5 years of diagnosis [2]. ALS has an estimated global prevalence of 2–3 per 100,000 individuals [3]. Its causes are heterogeneous, involving genetic [4], environmental [5], and epigenetic factors [6]. ALS is classified into sporadic ALS (sALS), which accounts for 90% of cases, and familial ALS (fALS), caused by inherited genetic mutations in approximately 10% of cases [7]. Key genetic contributors include mutations in the C9orf72 [8], SOD1 [9], TARDBP [10], and FUS genes [11]. Recent advances in genomics have identified over 50 genes associated with ALS [12].

Clinically, ALS exhibits significant phenotypic variability, encompassing both motor and non-motor manifestations. Motor phenotypes are defined by the distribution and progression of weakness, as well as the relative involvement of upper motor neurons (UMNs) and lower motor neurons (LMNs). The most common form, spinal-onset ALS, presents with asymmetric limb weakness and progresses to involve other regions, while bulbar-onset ALS starts with speech and swallowing difficulties. Rare phenotypes include flail arm and flail leg syndromes, characterized by proximal limb weakness, and primary lateral sclerosis (PLS), a UMN-dominant variant. Progressive muscular atrophy (PMA) involves isolated LMN degeneration. [13]. Non-motor symptoms, including behavioral changes [14], cognitive decline [15], and metabolic dysregulation [16], are observed in many patients, often contributing to a broader disease burden. Diagnosing ALS is challenging, due to symptom overlap with other neurological conditions, often resulting in delays [17]. Current diagnostic criteria rely on clinical assessment, genetic and laboratory tests, electromyography, and exclusion of ALS-mimicking conditions [18].

At the cellular level, ALS pathophysiology is widely recognized as multifactorial, involving a range of interconnected processes [19]. One of the primary pathological processes in ALS is glutamate-mediated excitotoxicity, where excessive glutamate accumulation leads to overactivation of motor neurons, resulting in cell damage and death [20]. Cortical hyperexcitability, an early and intrinsic feature of ALS, arises due to an imbalance between excitatory and inhibitory circuits in the motor cortex, particularly through a reduction in short-interval intracortical inhibition (SICI) mediated by GABAergic neurons [21]. This phenomenon contributes to the “dying-forward” hypothesis, which suggests that motor neuron degeneration begins in the cortex and progresses downstream to the spinal cord [22].

Mitochondrial dysfunction also plays a central role in ALS, as motor neurons rely heavily on uninterrupted ATP production [23]. Motor neurons are highly energy-dependent, and mitochondrial damage leads to impaired ATP production, increased oxidative stress, and the release of pro-apoptotic factors [24]. Oxidative stress further exacerbates cellular injury by damaging proteins, lipids, and nucleic acids [25]. Mutations in SOD1, a key antioxidant enzyme, can disrupt mitochondrial integrity and enhance oxidative damage [26].

Neuroinflammatory responses mediated by microglia, astrocytes, and other glial cells are other critical components in ALS progression [27]. Activated microglia and astrocytes release pro-inflammatory cytokines, chemokines, and reactive oxygen species (ROS), which exacerbate motor neuron damage [28]. These non-cell-autonomous mechanisms highlight the role of surrounding glial cells in motor neuron degeneration [29].

Locked-In State (LIS) and Completely Locked-In State (CLIS) are advanced stages of ALS characterized by severe motor impairments. In LIS, patients retain some voluntary eye movements or blinking, allowing limited communication, despite total paralysis of other voluntary muscles [30]. CLIS represents a progression from LIS, where all voluntary motor functions, including eye movements, are lost, rendering patients entirely unable to communicate through conventional means [31]. Both states result from extensive motor neuron degeneration, with CLIS reflecting near-complete cortical and corticospinal disconnection.

ALS is a complex disorder that causes numerous changes in the brain. To recognize, track, and understand their pathophysiology, brain neuroimaging methods are essential. Modern medicine knows several of these methods. Modern and advanced technologies include functional magnetic resonance imaging (fMRI) and positron emission tomography (PET). MRI and PET imaging reveal significant structural and functional brain changes that align with the disease’s pathological hallmarks. MRI studies, particularly those using diffusion tensor imaging (DTI), consistently demonstrate degeneration in corticospinal tracts and other white matter pathways, evidenced by reduced fractional anisotropy and increased mean diffusivity. These findings correlate with disease progression and motor dysfunction. Structural MRI also uncovers gray matter alterations, including hippocampal atrophy and cortical thinning, which are associated with cognitive and behavioral symptoms.

PET imaging, especially with ^18F-fluorodeoxyglucose (^18F-FDG), identifies distinct metabolic signatures in ALS. Hypometabolism is observed in the prefrontal, frontal, and motor regions, alongside hypermetabolism in the cerebellum and midbrain. These metabolic patterns distinguish ALS from other neurodegenerative diseases, with high accuracy. Furthermore, novel PET tracers targeting neuroinflammation, such as ^11C-PBR28, underscore increased glial activation in motor regions [32].

ALS is a complex disease that demands diverse approaches to investigate the brain’s abnormalities and changes. While advanced diagnostic tools have provided critical insights, they are often expensive and inaccessible. In contrast, electroencephalography (EEG)—one of the oldest and most fundamental methods of brain diagnostics—continues to be a valuable tool for studying ALS.

EEG is a non-invasive neuroimaging technique that records the brain’s electrical activity through electrodes placed on the scalp [33]. EEG provides a direct measure of neural activity and offers a unique window into the dynamic processes inside the brain by recording the voltage variations caused by the collective firing of neuronal populations [34]. EEG’s mobility and accessibility are significant additional benefits. EEG equipment is affordable and portable, and can be utilized at the patient’s bedside, whereas large MRI or PET scanners are expensive and stationary, and require patients to remain still in a confined space for extended periods. ALS patients, who may experience discomfort, mobility issues, or difficulty tolerating prolonged imaging sessions, particularly benefit from EEG. Furthermore, EEG can be conducted repeatedly, enabling the evaluation of treatment interventions over time and longitudinal monitoring of disease progression, because it records the brain’s natural electrical activity without exposing patients to radiation or other hazards.

To date, no review of studies has been published on EEG patterns in ALS. This review synthesizes existing evidence on resting-state EEG patterns in ALS patients, focusing on identifying consistent biomarkers and their underlying mechanisms. By integrating findings from multiple studies, it aims to elucidate the pathophysiological significance of altered EEG activity in ALS and evaluate its potential as a clinical marker. Additionally, the review offers recommendations for future research to address methodological gaps and improve the utility of EEG in ALS diagnostics and monitoring. While other neuroimaging modalities have contributed valuable insights into ALS pathophysiology, resting-state EEG holds a distinct and complementary role. Unlike task-based EEG, which captures brain activity during specific cognitive or motor tasks, resting-state EEG reflects baseline, intrinsic neural activity patterns that are not confounded by performance factors. This baseline perspective can be especially informative in ALS, where overt motor impairments may prevent patients from effectively engaging in tasks and where subtle cortical dysfunction often occurs even before clinical symptoms become pronounced.

Furthermore, resting-state EEG directly measures neural oscillations, providing a window into the underlying excitatory–inhibitory balance of cortical networks—processes intimately connected to ALS-associated cortical hyperexcitability. Its high temporal resolution enables the detection of transient network alterations and early pathophysiological changes that other neuroimaging techniques, which rely on slower metabolic or hemodynamic signals, might miss. The technique’s patient-friendly nature also facilitates repeated assessments over time, offering a potential biomarker for disease progression, response to therapy, and prognostic evaluations. In this context, a focused review on resting-state EEG patterns in ALS is both timely and needed, as it can synthesize existing evidence, highlight emerging trends, and clarify how resting-state EEG findings integrate with our broader understanding of ALS pathology.

## 2. Methods

Investigating resting-state EEG activity in ALS patients is the goal of this review. A thorough literature search and strict selection criteria were used to guarantee the validity and applicability of the findings. The approach focused on finding clinical trials and case studies that evaluated EEG activity in ALS and was guided by accepted procedures for conducting systematic reviews and evidence synthesis (PRISMA). However, as this is a mechanistic review rather than a systematic one, not all PRISMA methodology components were applied.

### 2.1. Data Sources and Search Strategy

J.Ch. and M.S.-S. conducted an independent, standards-based internet search using the following set of combined keywords while writing this review: “EEG” OR “electroencephalogram” OR “electroencephalography” AND “amyotrophic lateral sclerosis” OR “ALS”. Focusing on publications from January 1995 to October 2024, a comprehensive search was conducted in October 2024 across several databases, including PubMed/Medline, Research Gate, Google Scholar, and Cochrane. The cut-off date of January 1995 was selected to reflect advancements in EEG signal analysis methods and quality, avoiding the inclusion of older studies that might interfere with the synthesis of results.

### 2.2. Study Selection Criteria

To qualify for inclusion, publications had to be clinical trials or case studies published in English between January 1995 and October 2024. All non-English-language papers were excluded.

### 2.3. Screening Process

A number of screening procedures were implemented to ensure that relevant research was included and studies not meeting predefined criteria were excluded. In the initial screening step, abstracts and titles were carefully reviewed by two independent reviewers, J.Ch. and M.S.-S

#### 2.3.1. Title and Abstract Screening

Each reviewer independently assessed the abstracts and titles of the available records, to identify studies meeting the inclusion criteria. The primary focus of the screening criteria was the resting-state EEG in ALS.

#### 2.3.2. Full-Text Assessment

Following the initial screening of titles and abstracts, the selected papers were subjected to a detailed full-text review. The reviewers meticulously examined each publication to confirm that it met the eligibility requirements, with particular emphasis on whether the studies were clinical trials or case studies published in English between January 1995 and October 2024.

## 3. Results

**Figure 1:**
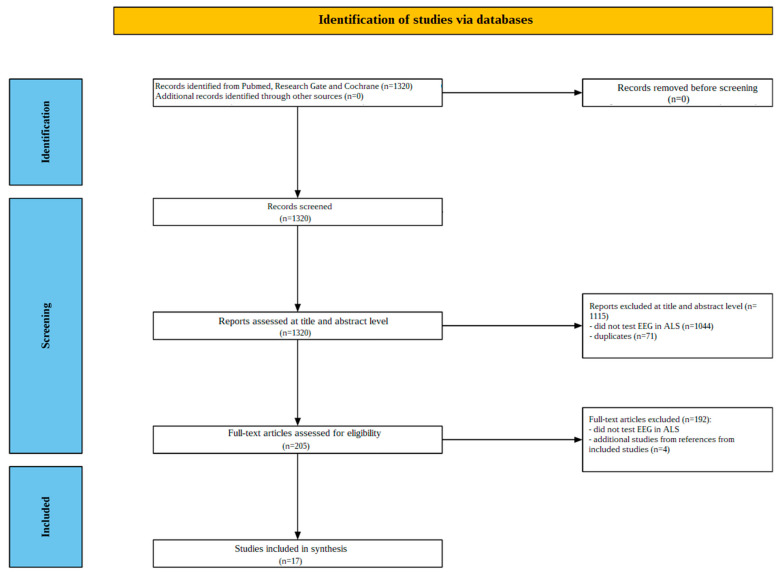
Flow chart depicting the different phases of the systematic review. This flow chart illustrates the phases of the systematic review, including the initial database search yielding 1320 studies, screening, exclusion, and the final inclusion of 17 studies. It highlights the number of records excluded at each stage and the final studies included for detailed analysis.

The screening procedure is depicted in Figure 1. Initially, database search results yielded 1320 studies. After reviewing the titles and abstracts, 1115 papers were excluded; 1044 of these studies did not investigate EEG in ALS, and 71 were duplicates. The remaining 205 papers underwent a thorough full-text analysis. Subsequently, 192 papers were disqualified for not examining the resting-state EEG in ALS. Thirteen studies were found to meet the inclusion criteria after a thorough review of their content. Additionally, bibliographies of studies that matched the review criteria were examined, identifying four additional relevant studies. Thus, a total of 17 studies were included in the review. These 17 studies [35,36,37,38,39,40,41,42,43,44,45,46,47,48,49,50,51] were published between 1998 and 2023.

### 3.1. Summary of Included Studies

The included studies are summarized in Table 1. In study [35], the goal was to clarify EEG’s potential as a severity measure for ALS. The authors used both spectral band powers and EEG microstates to characterize the brain’s spatiotemporal patterns activity during rest, and then correlated these aspects with clinical scores. Fifteen ALS patients (mean age 67.9 ± 10.9 years) and fifteen healthy controls (mean age 68.4 ± 8.0 years) participated in the study. Each patient underwent clinical evaluation at two time points: after recruitment (T0) and at 3 months (T1). Five minutes of EEG activity at rest with eyes closed were recorded by a 128-channel EEG system. The Power Spectral Density (PSD) was measured. Individual Alpha Frequency (IAF, 36) was used to define each of the analyzed frequency bands, separately: the slow frequency band, ranging from 1 Hz to 7 Hz (delta–theta); low alpha, ranging from IAF—2 Hz to IAF; high alpha, ranging from IAF to IAF + 2 Hz; and beta, ranging from 13 Hz to 25 Hz. Several clinical measures were recorded, including the ALS Functional Rating Scale—Revised (ALSFRS-R), the Manual Muscle Testing Score (MRC score), the Forced Vital Capacity (FVC), the ALS Assessment Questionnaire (ALSAQ-5), the King’s score (King), the MiToS score (MiToS), the Disease Progression score, and the Montreal Cognitive Assessment test (MoCA). Lower scores of ALSFRS-R and MRC for each leg at T1 suggested clinical deterioration, compared to T0. The patients’ mean MOCA score of 26 did not indicate significant cognitive impairment.

In study [36], the aim was to identify differences in EEG spectral power across four ALS phenotypes. The study included 95 ALS patients and 77 healthy controls (mean age: 60 ± 11). The ALS group consisted of 21 patients with bulbar-onset (60 ± 11 years), 4 patients with respiratory onset (62 ± 4 years), and 70 patients with spinal-onset (59 ± 12 years). A 6 min EEG recording was performed with eyes open. The six frequency bands were divided into ranges: δ (2–4 Hz), θ (5–7 Hz), α (8–13 Hz), β (14–30 Hz) and γ (γ_low_: 31–47 Hz, γ_high_: 53–97 Hz).

In study [37], EEG and functional near-infrared spectroscopy (fNIRS) were simultaneously used to measure brain activity. The study included 10 ALS patients (58.2 ± 11.6 years) and 9 healthy controls (61.0 ± 3.8 years). EEG recording lasted 5 min, and was performed with eyes closed. The power spectra for the following frequency bands were calculated: alpha (8.5–12.5 Hz), beta (12.5–30 Hz), theta (3.5–8.5 Hz), and delta (0.5–3.5 Hz).

In study [38] 6 ALS patients (mean age: 64 years) were included, and their results were compared to those of 37 healthy controls. A 5 min EEG recording was performed, with eyes open. The power spectra of each cortical source were calculated at the following frequency bands: δ (1–4 Hz), θ (4–8 Hz), α (8–14 Hz), β (20–30 Hz), γ_low_ (30–50 Hz), and γ_high_ (50–90 Hz).

In study [39], it was examined whether brain β-band oscillations in the sensorimotor network may function as a quantitative and objective indicator of increasing motor impairment and functional dysfunction in individuals with ALS. EEG data were collected from 18 ALS patients (mean age: 65.75) and 38 healthy, age- and gender-matched controls (66 years). Sensorimotor areas included six regions: left/right precentral, postcentral, and paracentral areas. The sensorimotor cortex’s source-localized β-band spectral power was calculated. The functional rating scale—revised for amyotrophic lateral sclerosis score, progression rate, fine motor function (FMF) subscore, and lower and upper motor neuron scores were included in the clinical evaluation. The EEG recording lasted 6 min, and was performed with eyes open. The beta band covered the frequency range of 14–30 Hz. A lower motor neuron (LMN) score and an upper motor neuron (UMN) score were used to grade motor impairment. Functional impairment was evaluated using the ALSFRS-R.

In study [40], 74 ALS patients were recruited. Fifty-six patients had spinal-onset (mean age: 57.9  ±  12.2 years), fifteen patients had bulbar-onset (59.0  ±  8.4 years), and three patients had respiratory onset (62.0  ±  5.3 years). The study also recruited 47 healthy controls (58.4  ±  12.3). The frequency bands are divided into six bands: delta (2–4 Hz), theta (5–7 Hz), alpha (8–13 Hz), beta (14–30 Hz) and gamma (γ_low_: 31–47 Hz, γ_high_: 53–97 Hz).

In study [41], eyes-open EEG was performed on 100 ALS patients (mean age: 60.2 ± 11.1 years), including 78 spinal-onset, 15 bulbar-onset, and 7 ALS–FTD cases. The control group consisted of 34 healthy individuals (58.1 ± 13.9 years). The frequency bands were divided as follows: δ (2–4 Hz), θ (5–7 Hz), α (α_low_: 8–10 Hz, α_high_: 11–13 Hz), β (β_low_: 14–20 Hz, β_high_: 21–30 Hz), and γ (γ_low_: 31–47 Hz, γ_high_: 53–97 Hz). The EEG recording lasted 6 min.

In study [42], EEG was measured with eyes closed in 21 people with ALS (mean age: 66 years). The control group consisted of 16 healthy people (65 years). The EEG recording lasted 5 min. The frequency bands were divided as follows: delta (1–4 Hz), theta (4–8 Hz), alpha (8–13 Hz), and beta (13–30 Hz).

In study [43], 17 healthy controls (mean age: 51 years) and 18 patients with non-familial ALS (56 years) were enrolled. There were 15 patients with spinal-onset, 2 with respiratory onset, and 1 with bulbar-onset. The EEG recording was performed with eyes open, and lasted 6 min. The frequency bands are divided as follows: delta (1–3 Hz), theta (4–7 Hz), alpha (8–13 Hz), low beta (14–21 Hz), high beta (22–30 Hz) and gamma (31–60 Hz).

Study [44] focused on understanding how EEG measures change over time in different phenotypes of ALS, particularly highlighting motor, cognitive, and behavioral impairments. The research analyzed EEG recordings from 124 ALS patients (mean age: 63.13 ± 15 years), categorized into subgroups based on cognitive impairment (ALSci = 27), behavioral impairment (ALSbi = 58), and no cognitive or behavioral impairment (ALSncbi = 53). The study revealed that longitudinal changes in EEG spectral power varied significantly across these phenotypes. The EEG recording lasted 6 min, and was performed with eyes open. The frequency bands were divided into δ (2–4 Hz), θ (4–7 Hz), α (7–13 Hz), β (13–30 Hz), low γ (30–47 Hz), and high γ (53–97 Hz).

In study [44], resting-state EEG with eyes closed was examined in people with ALS in a completely locked-in state (CLIS). The study included 10 patients with ALS (mean age: 47.1 years) and 7 healthy people (45.7 years). The frequency bands were divided as follows: delta (1−3 Hz), theta (4−7 Hz), low alpha (8−10 Hz), high alpha (11−13 Hz), beta (14−30 Hz), and gamma (31−40 Hz).

In study [46], EEG was examined in 18 ALS patients (mean age: 61.7 years) and 14 healthy individuals (63.4 years). Relative and absolute power were measured. Frequency bands were divided as follows: delta (1.5–4.0 Hz), theta (4.5–7.5 Hz), alpha (8.0–13.0 Hz), and beta (13.5–30 Hz).

Study [47] included 12 ALS patients (mean age: 46.75 years) and 9 controls (47.17 years). The patients had their eyes closed. Alpha power (8–13 Hz) was measured.

The purpose of study [48] was to examine the power spectral densities of EEG resting-state recordings over time in three ALS patients through a longitudinal analysis of EEG frequency. Out of the three individuals examined, two were in CLIS (P6 and P9), and the third was initially in the LIS-to-CLIS transition (P11), before ending up in CLIS. Patient P6 had bulbar ALS and was 40 years old, patient P9 had juvenile ALS and was 24 years old, and patient P11 had non-bulbar ALS and was 35 years old. The frequency range was divided into delta (0–4 Hz), theta (4–8 Hz), alpha (8–12 Hz), low beta (12–20 Hz), high beta (20–30 Hz), and gamma (30–45 Hz) bands. Relative power was measured with eyes closed. The observation period was 1 year.

Study [49] included four patients with ALS and CLIS. Patient P1 was 75 years old and had sporadic bulbar ALS; patient P4 was 29 years old and had juvenile ALS; patient P9 was 25 years old and had juvenile ALS; patient P17 was 63 years old and was diagnosed with ALS of the second motor neuron. Recordings were performed with eyes open and closed. No frequency-band range was specified.

Study [50] included 10 ALS patients (mean age: 51.5 years), including 2 in CLIS and 8 without CLIS. In addition, there was a control group consisting of 10 healthy individuals (61.4 years). The recording was performed with eyes open and closed.

In study [51], 13 LIS individuals (mean age: 51 years) and 15 cognitively normal control subjects (50.3 years) had their resting state, eyes-closed EEG data recorded. Patients were evaluated 2 to 23 months post diagnosis, reflecting both subacute and chronic phases of the syndrome. Delta (IAF-8 to IAF-6 Hz), theta (IAF-6 to IAF-4 Hz), alpha 1 (IAF-4 to IAF-2Hz), alpha 2 (IAF-2 to IAFHz), and alpha 3 (IAF to IAF+2Hz) were the bands of importance concerning the individual alpha frequency (IAF). Additionally, the beta 1 (13–20 Hz) and beta 2 (20–30 Hz) bands were analyzed.

### 3.2. Results of EEG Activity in Included Studies

Research by Notturno et al. [35] revealed that beta-band power in motor/frontal regions was found to be positively correlated with disease progression (higher disease progression scores), negatively correlated with clinical severity scores (lower global MRC score values), and higher in patients with higher disease burden. This correlation indicates that patients with a worse clinical state also exhibited higher beta power. Patients who experienced greater clinical deterioration at T1 compared to T0 also demonstrated increased beta power. When comparing groups with higher disease burden (King’s score = 4) to those with lower disease burden (King’s score < 4), beta power was significantly higher in the former (median [minimum–maximum]: 3.33 [2.19–3.60] vs. 2.31 [1.21–3.12], respectively). MiToS scores were similarly used to categorize patients, yielding consistent results. Although the patients’ median beta power was higher than that of healthy controls, the comparison between the higher-burden group and healthy controls did not reach statistical significance. No distinctions or patterns were observed when comparing patients with the least severity to healthy controls. Additionally, there was no significant variation in band powers between patients with UMN or LMN involvement. No discernible differences in IAF or spectral power were found between ALS patients and controls.

Investigations by Dukic et al. [36] indicated significant differences in EEG spectral power between ALS patients and healthy controls, as well as among different ALS subtypes. ALS patients exhibited distinct patterns in EEG spectral power, particularly in the β and γ bands, when compared to healthy controls. Four clusters of ALS patients were identified, based on EEG spectral features, each displaying unique neurophysiological profiles. Cluster 1 was characterized by increased beta-band spectral power in the frontotemporal network. Cluster 2 demonstrated increased alpha-band synchrony in the somatomotor network. Cluster 3 displayed a reduction in gamma-band synchrony in the frontotemporal network. Lastly, Cluster 4 exhibited heightened gamma-band comodulation in the frontoparietal network. The clinical profiles of the four ALS clusters highlighted specific patterns of functional, cognitive, and behavioral impairments that aligned closely with the distinct neurophysiological disruptions identified in the study. Cluster 1 was characterized by moderate limb motor impairment and mild deficits in verbal fluency, executive function, and memory, while language abilities were relatively preserved. These clinical features corresponded to the increased beta-band spectral power in the frontotemporal network. Cluster 2 exhibited mild motor dysfunction, preserved limb function, minimal bulbar symptoms, accompanied by mild language and memory deficits, but preserved executive function. In contrast, Cluster 3 displayed marked limb dysfunction and pronounced deficits in language, verbal fluency, and cognitive domains, particularly affecting memory and executive function. These severe impairments correlated with the decreased gamma-band synchrony in the frontotemporal network. Cluster 4 exhibited the most severe bulbar dysfunction and moderate limb impairment, alongside significant deficits in verbal fluency, executive function, and memory, as well as more pronounced behavioral impairments compared to other clusters. Survival analysis underscored the clinical relevance of these clusters. Cluster 2 had the longest survival (median ~6 years), reflecting milder motor and cognitive impairments, while Cluster 4 had the shortest survival (median ~3 years), consistent with severe network disruptions and clinical dysfunction. While statistical tests of clinical scores (e.g., ALSFRS-R, ECAS, BBI) did not reveal significant differences between clusters, observed trends aligned with neurophysiological profiles. Clusters with greater network disruptions, such as Clusters 3 and 4, were associated with more severe clinical impairments and shorter survival times.

Study [37] identified a significant reduction in EEG spectral power in ALS patients compared to healthy controls, particularly in the theta and alpha frequency bands. In the theta band, power reductions were prominent in frontal (e.g., F2, Fz), central (Cz), and temporal regions (e.g., T7, T8). For example, at channel T8, theta power was significantly lower in ALS patients (1.13 ± 0.84 µV^2^) compared to healthy controls (2.01 ± 0.91 µV^2^, *p* = 0.006), highlighting a robust decline in temporal region activity. Alpha-band power reductions were even more widespread, particularly in parietal and occipital regions. The most significant decrease was observed at channel PO8, where ALS patients exhibited an average power of 3.02 ± 0.75 µV^2^, compared to 5.84 ± 0.91 µV^2^ in healthy controls (*p* = 0.005). Significant reductions were also noted at channels P7, P8, and Oz.

Research in [38] reported a significant enhancement in global γ-band power in ALS, compared to healthy controls. This increase was observed across cortical independent components (ICs), and became more pronounced when reprojected onto electrodes, revealing spatial differences in mean band power. Specifically, ALS patients showed a notable trend of higher γ-band power across the brain and a peak of lower α-power in central regions of ALS patients. Furthermore, the study noted a significant reduction in γ-power in a patient with the most severe disease progression (ALSFRS-R score of zero).

Results from [39] showed that in the motor network, ALS patients exhibited significantly lower β-band power compared to healthy individuals. Higher β-band power was associated with greater muscular weakness in ALS patients, as determined by the LMN score, which demonstrated a strong correlation between the two variables. Additionally, a noteworthy association was observed between the β-band power and both the FMF subscore and FMF progression rate. Specifically, higher β-band power was linked to faster disease progression and greater functional impairment. However, the evidence did not support the hypothesis that lower β-power correlated with more severe UMN impairment.

Outcomes of study [40] indicated significant reductions in spectral power spanning δ- to β-bands. The most pronounced modifications were observed in the occipital and temporal (δ- to β-bands), lateral/orbitofrontal (δ- and θ-bands), and sensorimotor regions (β-band). The differences between ALS subgroups based on the pathologic hexanucleotide expansion in C9ORF72 and the place of onset (spinal, bulbar) were evaluated using EEG measurements that distinguished between ALS patients and healthy controls. The θ-band (L/R occipital gyri) and β-band (L/R post-central gyri and precuneus) achieved the highest AUC values for differentiating ALS patients from healthy controls.

The research in [41] highlighted significant EEG power alterations in ALS patients, particularly in the theta and gamma frequency bands. ALS patients exhibited reduced theta-band power over bilateral motor areas. In contrast, gamma-band power showed region-specific increases, particularly over frontoparietal regions.

Fraschini et al. [42] reported no significant variations across any frequency range in ALS patients compared to controls.

Study [43] found increased alpha-band power in the parietal region, elevated theta-band power in the central and frontal regions, and increased gamma-band power in the frontal, parietal, and occipital regions in ALS patients.

Key findings in research [44] revealed a significant decrease in θ-band spectral power over time, particularly in the temporal lobe, alongside an increase in γ-band spectral power in the frontal and temporal lobes. Subgroup-specific changes highlighted distinct patterns. Participants with behavioral impairment (ALSbi) exhibited an increase in γ-band spectral power specifically in the temporal lobe. Changes in θ-band and γ-band power negatively correlated with fine motor symptom progression, particularly in the ALSncbi group.

Analysis in study [45] revealed significant reductions in high alpha (11–13 Hz), beta (14–30 Hz), and gamma (31–40 Hz) frequency bands in CLIS patients. The most pronounced changes were observed in fronto-central (FC1, FC5, FC6) and central (Cz) sensors. CLIS patients exhibited a dominance of slower oscillations in the delta (1–3 Hz) and theta (4–7 Hz) bands, indicating a shift toward lower-frequency activity. Correlation analysis suggested a trend of decreasing power in higher-frequency bands with longer ALS duration, although this was not statistically significant.

Research [46] reported no significant difference in global EEG band power (all 16 electrodes) between ALS patients and control. However, regional EEG analysis showed a significant decrease in relative alpha power in the central areas. There were no discernible group differences in the posterior or frontal regions. The log-transformed relative beta, theta, and delta power for each electrode did not significantly differ. The relative alpha power for the C3, C4, and Cz electrodes demonstrated a substantial difference (a decrease in alpha activity), with a maximum over the C4 position. The groups also remained distinct across other electrodes.

Results in study [47] indicated that total alpha-band power in the left hemisphere was reduced by 60–70% in ALS patients compared to age-matched controls. While the alterations appeared bilateral, statistical analysis showed that the reductions were more significant in the left hemisphere (*p* ranging from 0.0007 to 0.0067) than in the right hemisphere (*p* ranging from 0.0030 to 0.1208). No significant differences in overall alpha were noted between the left and right hemispheres of ALS patients.

A paper [48] revealed that the EEG frequency content of CLIS patients 6 and 9 was shifted toward the delta and theta bands. No progression trends were observed over the monitoring period for these patients. Patient 11 exhibited consistent alpha band activity in all recordings; however, a marked reduction in the EEG signal strength occurred as the patient transitioned from LIS to CLIS. The EEG frequency content of patients 6 and 9 differed significantly from that observed in patient 11. The Mann–Whitney U test results showed a significant difference in the relative band power between patient 11 and CLIS patients 6 and 9 in the delta, alpha, and low-beta bands. Conversely, no differences were noted between patients 6 and 9.

Resting-state EEG findings in the research [49] revealed significant alterations in brain activity patterns among patients with CLIS, due to advanced ALS. The EEG showed pronounced slowing of frequencies, and severe attenuation or absence of alpha waves, typically observed in the range of 8–13 Hz. In two patients, a synchronized slow wave activity at approximately 4 Hz dominated across all EEG channels, with phase-synchronized waves appearing throughout the cortex.

Findings in study [50] highlighted significant slowing of the Alpha Peak Frequency (APF) in two CLIS ALS patients. APF values were 7 Hz for patient P1 and 3.8 Hz for patient P2, both below the typical alpha range of 8–13 Hz seen in healthy individuals and non-CLIS ALS patients. This slowing was accompanied by reduced activity in the typical alpha range and increased dominance of theta-range frequencies (3–7 Hz) and low alpha frequencies. Spectral analysis revealed that EEG patterns in CLIS patients differed significantly from those in healthy subjects and non-CLIS ALS patients, with the power distribution showing a mix of frontal theta activity and parietal alpha activity. This contrasted with healthy individuals and non-CLIS ALS patients, who exhibited prominent parietal alpha activity.

In research by Babiloni et al. [51], LIS patients had reduced alpha 2 and alpha 3 source power in all regions when compared to controls. Conversely, patients with LIS had greater delta source power in central, parietal, occipital, and temporal regions compared to controls.

## 4. Discussion: Frequency-Specific EEG Changes in ALS

In this review, we analyzed 17 studies examining resting-state EEG activity in ALS, focusing on the relationships between spectral power in different frequency bands (delta, theta, alpha, beta, and gamma) and disease severity, clinical presentation, and potential neurophysiological mechanisms. Across multiple cohorts, significant alterations were observed in alpha and beta power, with some studies associating increased beta activity with advanced clinical severity. Additionally, a shift toward lower-frequency bands (delta and theta) was noted in late-stage or completely locked-in state (CLIS) patients, indicating profound cortical dysfunction. However, findings on theta power, alpha asymmetry, and gamma-power changes varied. depending on the study population and disease stage. This variability underscores the clinical and neurophysiological complexity of ALS, as well as the diverse methods used in EEG research. The following subsections provide a detailed analysis of the findings for each frequency band.

### 4.1. Delta Activity

Findings regarding delta-band (~1–4 Hz) activity in ALS patients are heterogeneous, varying with the clinical stage of the disease and the specific ALS subtype or phenotype. Some studies found no significant differences in delta activity between ALS patients and healthy controls. However, other studies reported alterations in delta power, especially in advanced or locked-in states (LIS/CLIS), where pronounced cortical slowing is observed.

Several studies focusing on earlier or less severe ALS cases did not find marked differences in delta power relative to controls. For instance, in investigations emphasizing beta- and gamma-band abnormalities, delta activity often appeared relatively preserved. Similarly, studies on patients with predominantly limb or bulbar-onset ALS, without advanced clinical severity, often showed minimal or no significant differences in delta power. In these cases, the most prominent EEG biomarkers appeared in higher-frequency bands (theta, alpha, beta, gamma), reflecting changes in corticospinal and cognitive domains rather than a fundamental shift toward lower-frequency rhythms. Thus, delta power per se was not consistently linked to mild-to-moderate disease severity or specific ALS subtypes at early disease stages.

In advanced ALS, including those in the locked-in state or completely locked-in state, multiple studies demonstrated a significant slowing of the resting-state EEG activity. This slowing often manifests as a pronounced increase in lower-frequency bands, including delta. In these stages, the cortical rhythms are dominated by slower oscillations (delta and theta), reflecting severe motor impairment, progressive cortical dysfunction, and the loss of normal alpha rhythms. For example, studies on ALS patients transitioning to, or already in, CLIS often noted a shift in the EEG spectrum toward delta and theta frequencies, accompanied by diminished power in faster oscillations. This shift toward lower-frequency bands is accompanied by the attenuation or near absence of alpha activity and has been proposed as a neurophysiological correlate of end-stage cortical disconnection and reduced sensorimotor interaction with the environment.

While some investigations reporting delta activity changes did not provide a detailed regional analysis, those that did highlighted the involvement of sensorimotor and fronto-central regions. In CLIS patients, delta activity, along with theta power, dominates over large portions of the cortex. In LIS, increased delta power has been observed in central, parietal, occipital, and temporal regions, compared to controls. These alterations often coincide with marked reductions in faster frequencies, reflecting global cortical reorganization and a transition from an active, responsive neural network to a more synchronized, low-frequency state. This shift is likely associated with the loss of voluntary motor output and diminishing responsiveness.

The relationship between delta activity and clinical measures of ALS progression is complex. At earlier stages, no consistent correlation between delta power and conventional clinical scales (e.g., ALSFRS-R) or cognitive status has been firmly established. However, in advanced disease states, the emergence of dominant delta activity corresponds to severe motor impairment and cortical disconnection, which parallels declining clinical function. Although strong statistical associations were not always reported, the pattern of slower oscillations aligns with the known pathophysiology of ALS, including near-complete loss of voluntary motor capabilities and communication pathways.

### 4.2. Theta Activity

Theta-activity results across the reviewed studies revealed a complex and sometimes contradictory picture of how theta-band EEG oscillations are affected in patients with ALS. For instance, study [37] demonstrated notable reductions in theta-band power in ALS patients, particularly in frontal (F2, Fz), central (Cz), and temporal regions (T7, T8). At channel T8, ALS patients showed a theta power of 1.13 ± 0.84 µV^2^ compared to 2.01 ± 0.91 µV^2^ in controls (*p* = 0.006), highlighting a significant decline in temporal activity. Similarly, study [40] reported that spectrum power reductions in ALS patients extended from the delta to the beta band, with significant modifications in the occipital and temporal regions. Theta-band power in the left and right occipital gyri achieved a high area under the curve (AUC) values, distinguishing between ALS patients and healthy controls, and indicating notable reductions. Study [41] also found marked reductions in theta-band power over bilateral motor areas in ALS patients compared to controls, emphasizing alterations in motor network oscillations.

Contrastingly, other studies reported increases in theta activity among ALS patients. Study [43] observed elevated cross-spectral density in the theta band, particularly in parietal regions, with significant increases across all scalp areas, especially in the frontal and central areas. Additionally, study [44] found an overall decrease in theta power over time across all ALS patient groups. However, patients with cognitive impairment (the ALSci subgroup) showed increased theta-band co-modulation, which was correlated with declines in verbal fluency and overall cognitive scores. This increase suggesting that heightened theta activity may be associated with cognitive deterioration in ALS. Elevated theta activity in these contexts may indicate compensatory mechanisms or pathological overactivity in response to neuronal damage.

In advanced ALS stages and in patients with severe impairments, such as those in a completely locked-in state (CLIS), theta activity shows distinct patterns. Study [45] reported that the EEG of CLIS patients was dominated by slower oscillations, including the theta band, indicating a shift toward lower-frequency activity and suggesting extensive cortical dysfunction. Study [48] showed that CLIS patients exhibited a frequency content shifted toward the delta and theta bands, with no overall trend progression over the monitoring period. This dominance of slower frequencies may reflect the severity of cortical impairment in advanced disease stages. Similarly, study [49] noted synchronized slow-wave activity around 4 Hz, at the borderline of the theta range, dominating EEG channels in some CLIS patients, indicating a pronounced slowing of brain activity.

Some studies found no significant differences in theta activity between ALS patients and healthy controls. Studies [35,42,46] reported no discernible changes in theta-band power, suggesting that theta activity may not be uniformly affected across all ALS patients and may depend on disease stage or individual variability. Study [36], which focused on beta and gamma bands among ALS phenotypes, did not report findings related to theta activity.

Theta activity may also vary with disease progression or specific ALS phenotype. Study [50] showed increased dominance of theta-range frequencies in CLIS patients, alongside significant slowing of the alpha peak frequency. This shift, accompanied by reduced typical alpha activity, distinguished CLIS patients from non-CLIS ALS patients and healthy controls. While study [51] primarily noted increased delta power and decreased alpha power in LIS patients, it implies that theta activity may also be affected, though specific theta findings were not highlighted.

### 4.3. Alpha Activity

Multiple studies have reported significant reductions in alpha-band power among ALS patients, compared to healthy controls. Study [37] observed widespread reductions in alpha power in ALS patients, particularly in the parietal and occipital regions during eyes-closed resting-state EEGs. The most notable decrease was at channel PO8, where ALS patients had significantly lower alpha power (3.02 ± 0.75 µV^2^) compared to controls (5.84 ± 0.91 µV^2^, *p* = 0.005). Similarly, study [38] identified a trend of reduced alpha power in the central regions of ALS patients, accompanied by a global increase in gamma-band power, indicating altered cortical excitability. Study [46] reported significant reductions in relative alpha power over central regions (C3, C4, Cz electrodes) during eyes-closed EEGs, with no significant differences in other frequency bands or regions, highlighting localized alterations in alpha activity. Study [47] demonstrated a substantial reduction, between 60% and 70%, in total alpha-band power in the left hemisphere of ALS patients compared to age-matched controls. While reductions were bilateral, they were more pronounced on the left side (*p*-values ranging from 0.0007 to 0.0067), suggesting hemispheric asymmetry in cortical dysfunction.

Patients in advanced stages of ALS, such as those in CLIS or LIS, exhibit pronounced alterations in alpha activity. Study [45] reported significant reductions in high alpha (11–13 Hz) power in CLIS patients, particularly over fronto-central regions (FC1, FC5, FC6, Cz). These EEGs were dominated by slower oscillations, reflecting a shift toward lower-frequency activity. Study [48] observed a shift in EEG frequency content toward delta and theta bands in CLIS patients over a one-year longitudinal study. Patient 11, transitioning from LIS to CLIS, showed a discernible drop in alpha-band activity, correlating with disease progression. Study [49] reported a pronounced slowing of frequencies in CLIS patients, with a notable absence or severe attenuation of alpha waves. In some patients, synchronized slow-wave activity around 4 Hz dominated the EEG, indicating severe cortical impairment. Study [50] highlighted a significant slowing of the alpha peak frequency in CLIS patients, with values below the typical 8–13 Hz range (7 Hz and 3.8 Hz in two patients). This slowing was accompanied by reduced activity within the typical alpha range and increased dominance of theta-range frequencies. Study [51] found that LIS patients had reduced alpha 2 and alpha 3 source power across all cortical regions, compared to controls. The decrease in higher alpha frequencies was accompanied by increased delta power in central, parietal, occipital, and temporal regions.

In contrast to studies showing decreased alpha power, some research identified increased alpha activity or synchrony in certain ALS subgroups. Study [36] identified a subphenotype of ALS patients (Cluster 2) characterized by increased alpha-band synchrony within the somatomotor network during eyes-open EEGs. Clinically, these patients exhibited mild motor dysfunction with preserved limb function, minimal bulbar symptoms, and mild language and memory deficits. Research [43] reported increased cross-spectral density in the alpha band (8–13 Hz) in ALS patients, particularly in parietal regions, during eyes-open EEG recordings. This finding suggests enhanced connectivity or compensatory mechanisms in specific cortical areas.

Some studies did not find significant differences in alpha activity between ALS patients and healthy controls. Study [35] reported no discernible variation in individual alpha frequency or spectral power, including the alpha band, between ALS patients and controls during eyes-closed EEG recordings. Study [42] similarly reported no significant differences across any frequency band, including alpha, between ALS patients and controls during eyes-closed EEGs, suggesting that alpha activity may remain unchanged in certain patient populations.

Several studies have identified correlations between alpha activity and clinical measures in ALS patients, suggesting that changes in alpha-band power and synchrony reflect disease severity, progression, and motor or cognitive function. In advanced stages of ALS, such as in patients who are completely locked-in (CLIS) or in the LIS, significant reductions in alpha power have been observed, correlating with disease severity. For instance, study [45] found significant reductions in high alpha (11–13 Hz) power over fronto-central regions in CLIS patients. These reductions were accompanied by the dominance of slower oscillations, indicating a shift toward lower-frequency activity associated with severe motor impairment. Similarly, study [48] observed a decline in alpha-band activity as a patient transitioned from LIS to CLIS over a one-year period. This reduction correlated with disease progression, highlighting alpha power as a potential marker for advancing disease.

The slowing of the Alpha Peak Frequency (APF) has also been noted to correlate with disease severity. Study [50] reported a significant slowing of the APF in CLIS patients, with values falling below the typical 8–13 Hz range (7 Hz and 3.8 Hz). This slowing was associated with reduced alpha-band activity and increased dominance of theta-range frequencies, reflecting cortical dysfunction associated with disease severity.

Correlations with motor function have also been observed. Increased alpha synchrony has been associated with milder motor impairment. Study [36] identified a subgroup of ALS patients characterized by increased alpha-band synchrony within the somatomotor network. Clinically, these patients exhibited mild motor dysfunction, preserved limb function, and minimal bulbar symptoms, suggesting that enhanced alpha synchrony may represent compensatory mechanisms or less severe motor neuron degeneration. Additionally, hemispheric asymmetry in alpha power has been linked to motor dysfunction. Study [47] found a significant decrease in alpha power, which was more pronounced in the left hemisphere. While the study did not directly correlate this asymmetry with motor function, the left hemisphere is often associated with language and fine motor skills, implying potential links to motor impairment.

In terms of cognitive function, reductions in alpha activity have been correlated with cognitive impairments. Research [51] reported that patients with LIS had reduced alpha 2 and alpha 3 source power across all cortical regions, accompanied by increased delta power. These changes correlated with cognitive deficits often observed in advanced ALS, suggesting that decreased alpha activity may reflect cognitive decline.

Reduced alpha power also occurs in other neurodegenerative diseases. Some review articles demonstrate that reduced alpha power is a significant hallmark of Alzheimer’s disease (AD), and often manifests in Mild Cognitive Impairment (MCI), the prodromal stage of AD. This reduction in alpha power is a critical aspect of the EEG slowing phenomenon commonly observed in AD and MCI, where brain rhythms shift from faster frequencies, such as alpha and beta, toward slower frequencies, such as delta and theta [52,53].

In individuals with MCI, reductions in alpha power are present, but tend to be less pronounced than in AD, often localized to specific brain regions associated with memory and executive functions. This localized reduction supports the notion that MCI represents an intermediate stage in the continuum of cognitive decline, where changes in brain activity begin to emerge but are not as widespread or severe as in AD. For instance, reductions in alpha power in posterior regions of the brain have been linked to early-stage disruptions in memory networks, a key feature of MCI [54].

### 4.4. Beta Activity

Across the studies, beta-band (approximately 13–30 Hz) EEG activity in ALS patients emerges as a complex and context-dependent marker that correlates with disease severity, clinical subtypes, and patterns of neurophysiological impairment. Several studies highlight a link between elevated beta power and more severe ALS phenotypes. For instance, Notturno et al. [35] found that higher beta power in motor/frontal regions was positively correlated with disease progression scores and negatively correlated with strength measures (MRC scores), indicating that patients with greater clinical deterioration tended to exhibit higher beta-band activity. Similarly, study [39] demonstrated that within ALS groups, greater beta-band power in sensorimotor areas was associated with more pronounced muscle weakness, faster functional decline, and higher progression rates. These findings suggest that heightened beta activity can serve as a marker of worsening motor function and advancing disease.

However, it is important to note that while increases in beta power within ALS patients may signal greater impairment, ALS populations as a whole sometimes show altered or even reduced beta power, when compared to healthy controls. Studies [40,44,45] indicate that ALS, particularly in advanced stages like the CLIS, can be characterized by overall reductions in power or connectivity in the beta band. In such cases, beta activity, along with other higher-frequency bands, diminishes substantially, reflecting profound cortical dysfunction as ALS severity deepens to end-stage conditions.

The relationship between beta activity and ALS is not uniform across all patients; instead, distinct ALS phenotypes exhibit unique beta-band signatures. For example, in study [36], one ALS subphenotype (Cluster 1) showed increased beta power in the frontotemporal network, accompanied by mild cognitive and motor deficits. Conversely, other clusters with different clinical profiles did not display beta elevations, underscoring the potential utility of beta patterns in differentiating neurophysiological subtypes.

Longitudinal analyses and subgroup-based investigations, such as those in study [44], reveal further complexities. ALS patients with cognitive impairment (ALSci) exhibited widespread increases in co-modulation across delta, theta, and beta bands, linking heightened beta interactions to cognitive decline. In contrast, ALS patients without cognitive or behavioral impairments (ALSncbi) showed widespread reductions in beta-band synchrony, which were associated with motor decline. These findings suggest that beta-band alterations may map onto distinct clinical trajectories: increases in beta connectivity in frontotemporal areas may signal cognitive deterioration, whereas decreases in sensorimotor beta synchrony often accompany progressive motor impairment.

Beta abnormalities are frequently region-specific. Motor and sensorimotor areas feature prominently, particularly when beta power correlates with muscle weakness or motor scores [35,39,40,44]. In some cases, frontal and frontotemporal networks exhibit characteristic beta changes that align with cognitive and executive deficits [35,36,44]. These region-specific patterns demonstrate that beta activity is not a uniform measure of disease burden, but reflects localized or network-level cortical dysfunctions associated with clinical symptoms.

The progression of beta activity as ALS worsens is another recurrent pattern. Advanced stages, such as CLIS, often show reduced beta, along with a general slowing of EEG frequencies, whereas early or moderate stages may present relatively intact or even enhanced beta power, linked to declining motor function [45,48]. As cortical injury becomes more substantial, the brain’s ability to sustain higher-frequency oscillations diminishes, leading to a shift from aberrant beta rhythms to a state dominated by slower frequencies.

Not all investigations have found robust changes in beta activity. Some studies reported no significant differences between ALS patients and controls [42,46] or no distinct patterns within certain subgroups. These inconsistencies may stem from variations in sample populations, recording conditions (e.g., eyes open vs. closed), disease stage, or phenotypic expression. Nonetheless, the broader literature generally supports the notion that beta activity often diverges in ALS patients compared to healthy individuals, and its alterations carry clinical significance.

### 4.5. Gamma Activity

Gamma activity, typically defined as oscillations above 30 Hz, shows complex and stage-dependent changes in ALS. While not all studies examined gamma bands, those that did reported both increases and decreases in gamma power. Several studies [36,38,41,43,44] observed elevated gamma-band activity in ALS patients compared to controls or certain subgroups. ALS clusters or subgroups with less severe clinical impairment often display elevated gamma, which is associated with slower disease progression.

However, in end-stage conditions such as CLIS, gamma power tends to decline [45]. Gamma activity in ALS exhibits a complex pattern influenced by disease stage, phenotype, and the presence of cognitive or behavioral impairments. While early and intermediate phases may display increased gamma power or synchrony, gamma activity progressively declines as ALS advances to more severe stages, such as CLIS.

## 5. Pathophysiological Mechanisms of ALS Based on Consistent EEG Findings

**Figure 2:**
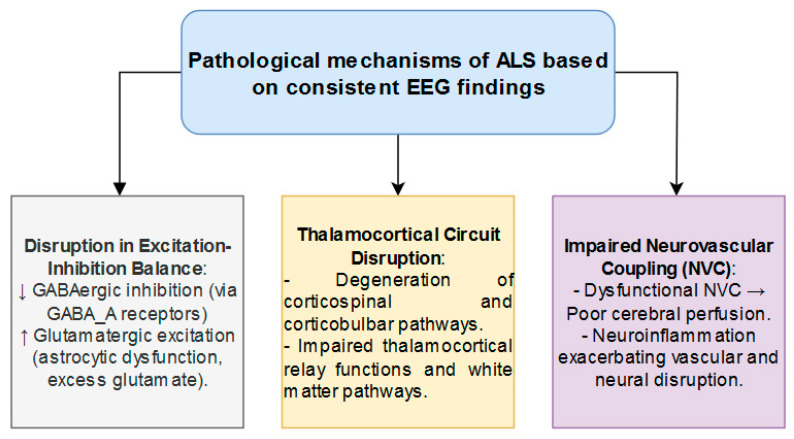
Pathophysiological mechanisms of ALS based on consistent EEG findings. This figure depicts the key pathophysiological mechanisms underlying ALS as revealed by consistent findings from resting-state EEG studies. It provides a conceptual framework linking EEG biomarkers to ALS pathophysiology.

A fairly consistent picture emerges from the studies discussed in this mechanistic review: ALS is associated with decreased EEG alpha power. Exploring the possible pathophysiological mechanisms behind this finding, even if hypothetical, may lead to the discovery of new treatments and validate existing ones. Furthermore, conducting EEG studies in patients with ALS and detecting patterns similar to those identified here may encourage the widespread use of tailored therapies. A graphical representation of the mechanisms is presented in Figure 2.

### 5.1. Alpha Oscillations as a Marker of Disrupted Cortical Excitability in ALS

Alpha oscillations, ranging from 8 to 12 Hz, are predominant in the resting brain and play an essential role in sensory processing [55], attention [56], and the coordination of neural networks [56,57]. These rhythms are generated by thalamocortical and cortico-cortical circuits [58,59] and rely on a balance between excitatory (glutamatergic) and inhibitory (GABAergic) signaling [60]. Key neural processes driving alpha oscillations include GABAergic inhibition mediated by interneurons through GABA_A receptors, which regulate the firing of pyramidal neurons and prevent excessive excitatory activity [60], and regulated glutamatergic excitation, ensuring optimal neural responsiveness.

GABAergic interneurons, particularly those acting through GABA_A receptors, are essential for the generation and maintenance of alpha power [61]. Experimental evidence shows that pharmacological enhancement of GABAergic signaling boosts alpha oscillatory activity [60], whereas disruptions in GABAergic function or excessive glutamatergic activity reduce alpha power [60]. Moreover, thalamic contributions play a pivotal role in synchronizing cortical alpha rhythms, with the pulvinar nucleus acting as a key modulator of thalamocortical coherence [60].

This inhibitory control can be measured via short-interval intracortical inhibition (SICI) using transcranial magnetic stimulation (TMS), serving as an indicator of GABA_A receptor function [62]. Disruption of SICI is associated with diminished alpha activity, underscoring the importance of inhibitory mechanisms in maintaining neural synchrony. On the excitatory front, glutamate-mediated inputs contribute to oscillatory dynamics by providing a counterbalance to inhibition [62]. However, excessive glutamatergic activity overwhelms inhibitory control, destabilizing alpha oscillations and leading to disrupted rhythms and reduced alpha power. This imbalance, favoring excitation over inhibition, is a hallmark of several neurological conditions, and often manifests as diminished alpha activity.

ALS exemplifies the imbalance between excitation and inhibition, characterized by progressive motor neuron degeneration and early-onset cortical hyperexcitability [63,64]. A significant reduction in GABAergic inhibitory signaling within the motor cortex, particularly involving interneurons acting via GABA_A receptors, is a hallmark of ALS pathophysiology [63]. This loss of inhibitory control is evidenced by decreased SICI, which reflects impaired regulation of neural excitability [65]. This dysfunction also leads to reductions in alpha oscillatory activity, as robust GABAergic signaling is essential for the stability and synchronization of these rhythms [60]. Studies have shown that diminished GABAergic function results in weaker and less synchronized alpha oscillations [60], establishing a mechanistic overlap between ALS-related cortical dysfunction and impaired neural synchrony.

At the same time, heightened glutamatergic excitatory activity is another defining feature of ALS. Astrocytic dysfunction compromises the clearance of extracellular glutamate through excitatory amino acid transporters (EAATs) [66], leading to glutamate accumulation and excitotoxicity [64]. This overactivation of glutamate receptors destabilizes neural circuits, exacerbating motor neuron vulnerability and disrupting the synchrony required for alpha oscillations. Pharmacological studies reveal that glutamate receptor overactivation decreases alpha power [60], further linking ALS-related excitotoxicity to impaired oscillatory activity.

Glial cell dysfunction, particularly involving microglia and astrocytes, plays a critical role in the progression of ALS [64] and may contribute to both reduced alpha oscillations and cortical hyperexcitability. Dysregulated microglia release pro-inflammatory cytokines, exacerbating neuronal damage and impairing GABAergic and glutamatergic signaling [64]. Similarly, astrocytes, which are normally responsible for glutamate recycling, fail to maintain neurotransmitter homeostasis, leading to unchecked glutamate accumulation and further excitotoxicity [64]. These disruptions amplify the excitatory state and diminish inhibitory support, destabilizing the neural circuits responsible for generating synchronized alpha rhythms.

Oxidative stress and neuroinflammation further exacerbate the disruption of alpha rhythms in ALS. Reactive oxygen species (ROS) and inflammatory cytokines released by activated glial cells cause additional damage to neurons and glial cells [66]. These processes interfere with the regulatory systems that maintain neural excitability and synchrony [66], and may lead to a further decrease in alpha oscillatory power. The combined effects of oxidative stress and neuroinflammation create a self-reinforcing loop of neural dysfunction that impairs cortical stability.

Overall, the mechanistic overlap between reduced alpha activity and cortical hyperexcitability in ALS lies in their shared disruption of the excitation–inhibition balance. Both conditions stem from decreased GABAergic inhibition and increased glutamatergic excitation. Impaired GABA_A receptor function and excess glutamate disrupt neural synchrony, affecting the neural circuits responsible for generating alpha oscillations and leading to destabilized cortical activity. This mirrors the hyperexcitability observed in the ALS motor cortex, perpetuating a self-reinforcing loop of neural dysfunction.

### 5.2. Loss of Thalamocortical Interactions

In ALS, the reduction in alpha power is closely linked to disruptions in thalamocortical interactions. The thalamus plays a pivotal role in generating and regulating alpha oscillations through its reciprocal connections with the cortex [67]. These thalamocortical circuits are essential for synchronizing neuronal activity and facilitating coherent alpha rhythms [68]. Studies suggest that ALS-related neurodegeneration disrupts these circuits. For instance, the loss of corticospinal and corticobulbar pathways, which impacts cortical excitation, also interferes with thalamocortical relay functions [69]. This degeneration impairs the dynamic feedback between the thalamus and cortex, resulting in diminished alpha oscillatory activity [70].

Furthermore, the degeneration of white matter pathways connecting the thalamus and cortical regions, as shown in diffusion tensor imaging (DTI) studies [71], leads to decreased network efficiency and impaired alpha power. Reduced input from the thalamus to cortical areas directly affects the generation of alpha rhythms [68]. Additionally, the thalamocortical network’s ability to sustain alpha-range oscillations relies on balanced input connectivity [72]. Alterations in this balance, as observed in ALS, can impair the generation and maintenance of alpha rhythms.

### 5.3. Impaired Neurovascular Coupling

Impaired neurovascular coupling (NVC) may be a significant factor contributing to the reduction of alpha power in ALS patients. NVC refers to the relationship between neuronal activity and subsequent changes in cerebral blood flow, which ensures that active brain regions receive adequate oxygen and nutrients [73]. In ALS, this coupling is frequently disrupted. Studies utilizing functional near-infrared spectroscopy (fNIRS) have observed that ALS patients exhibit abnormal hemodynamic responses during cognitive tasks [74]. Decreased alpha power in ALS patients is associated with impaired NVC, as the brain’s ability to modulate blood flow in response to alpha oscillatory activity is compromised. The weakened alpha rhythms reflect disrupted neural synchrony and connectivity, particularly in motor and frontoparietal networks.

Furthermore, ALS is characterized by neuroinflammatory processes that can damage the vascular endothelium, leading to blood–brain barrier disruption and impaired cerebral perfusion. This vascular dysfunction further exacerbates NVC impairments.

## 6. Clinical Implications of Findings from the Review

The shared mechanisms underlying alpha oscillation deficits and ALS pathophysiology suggest potential therapeutic targets. Enhancing GABAergic inhibition, either through pharmacological agents or neuromodulatory interventions, could help restore alpha power and reduce cortical hyperexcitability. Similarly, strategies to mitigate glutamate excitotoxicity (e.g., enhancing astrocytic glutamate uptake or using glutamate receptor antagonists) may help stabilize neural networks. These measures could also improve alpha rhythm synchronization. Targeting glial dysfunction and reducing oxidative stress could further alleviate disruptions in the excitation–inhibition balance. This multifaceted approach holds potential to address both ALS progression and its impact on cortical rhythms.

One promising therapeutic strategy is neurofeedback, a psychophysiological approach that enables individuals to learn to self-regulate specific brain functions by receiving real-time feedback on their neural activity. EEG (electroencephalography) and fMRI (functional magnetic resonance imaging) are commonly used to monitor neural activity, with the information presented visually, audibly, or through other sensory modalities. By learning to control neural impulses, individuals can influence brain activity in specific networks or regions, potentially improving behavior and cognitive abilities, and alleviating symptoms of certain neurological disorders. Unlike traditional neuroimaging (which identifies associations between brain activity and behavior), neurofeedback facilitates voluntary control of neural substrates, allowing researchers to explore causal relationships more directly. This self-regulation is accomplished through a learning process akin to operant conditioning, in which desired brain responses are reinforced through contingent feedback. Neurofeedback offers potential therapeutic benefits for neurological and psychiatric disorders, while providing fresh perspectives on neuroplasticity and the connections between the brain and behavior [75].

The use of neurofeedback in ALS was investigated in one study in this review. In study [76], the target frequencies included sensorimotor rhythm (SMR, the so-called high alpha, 12–15 Hz)**,** theta (4–8 Hz), and beta2 (frequencies above 18 Hz)**.** The goal was to increase SMR waves to reduce impulsive behavior and enhance relaxation. At the same time, the amplitude and percentage of theta and beta2 waves were reduced, to mitigate anxiety and psychomotor agitation. The results of the study showed significant improvements following the neurofeedback intervention. After 10 sessions, there was a notable increase in the amplitude of SMR waves in the right hemisphere and a desired reduction in beta2 wave activity. These neurophysiological changes coincided with improvements in the patient’s mental state, including mood stabilization, greater motivation for rehabilitation, and enhanced concentration. These factors helped the patient engage more fully in physical therapy. As a result, physical rehabilitation outcomes also improved, with increased muscle strength in the upper and lower limbs, postural stability, movement coordination, and fine motor skills. Pain levels were reduced from 10 to 3 points on the VAS scale, and walking distance increased from 192 m to 224 m using a walker.

Given that ALS patients often exhibit reduced alpha power, neurofeedback protocols should aim to enhance this frequency range. Expanding the range of alpha training to frequencies from 8 to 13 Hz, which includes the SMR rhythm, may provide comprehensive benefits for ALS patients.

## 7. Methodological Limitations in EEG Studies in ALS and Strategies for Improvement

### 7.1. Relative vs. Absolute Power

A significant methodological limitation in EEG studies of ALS is the inconsistent use of relative power versus absolute power to measure spectral activity across frequency bands. Both measures provide different insights into brain oscillatory activity, but their mixed application complicates comparisons and generalizability across studies.

Absolute power refers to the raw power values within a specific frequency band, directly quantifying the strength of the EEG signal without normalization. This measure provides a clear representation of oscillatory activity [77]. For instance, studies [37,39] reported absolute power reductions in alpha and theta bands in ALS patients. Because absolute power is less affected by changes in other frequency bands, it is a more precise measure for identifying specific alterations.

Relative power, on the other hand, is the proportion of power in a specific frequency band relative to the total power across all bands. It normalizes the spectral power, to account for individual variability in EEG amplitude [77]. While relative power is useful for controlling inter-individual variability, it may obscure changes in specific frequency bands if power reductions or increases occur unevenly across the spectrum. For example, study [46] reported relative alpha power differences in ALS patients, but global EEG data based on absolute power did not show significant group effects. This discrepancy highlights the limitations of relying solely on relative measures.

In ALS, global EEG power often declines, reflecting a slowing of cortical activity. As a result, relying on relative power can obscure important findings in specific bands, especially when slower frequencies (delta and theta) rise at the expense of alpha and beta rhythms. For example, a decline in absolute alpha power may appear less pronounced when normalized against increased delta power, leading to the underestimation of disease-related disruptions.

To address this limitation, future studies should report both absolute and relative power for all frequency bands, to enable more comprehensive analysis and cross-study comparisons. Researchers should explicitly clarify the rationale for using one measure over the other, particularly when interpreting results in the context of ALS progression and cortical dysfunction. Additionally, researchers should prioritize absolute power when assessing localized cortical changes. This approach eliminates normalization biases and offers a more accurate measure of neural oscillatory strength. By addressing these inconsistencies, future studies can better identify specific spectral-power changes in ALS, and enhance the reliability and comparability of EEG findings across research groups.

### 7.2. Age Differences

Differences in the age of ALS patients and control participants represent another critical methodological limitation in EEG studies, as aging significantly affects brain oscillatory activity. EEG power in various frequency bands naturally declines with age in the alpha [78] range, while oscillations such as beta may increase [79]. Some research has linked healthy aging to increases in theta (4–7.5 Hz) and delta (1–4 Hz) power, whereas other studies have found declines in these parameters [80]. If age differences between ALS patients and controls are not accounted for, it becomes difficult to disentangle the effects of ALS pathology from age-related neural changes.

Study [40] demonstrated good age-matching between ALS patients (mean age: 57.9 ± 12.2 years) and controls (58.4 ± 12.3 years), reducing potential bias. However, other studies, such as [37,43], included ALS patients with mean ages in the late 50s to 60s, while recruiting younger controls (e.g., 51 years), potentially introducing confounding effects. These disparities are particularly problematic when analyzing alpha power, where natural age-related reductions overlap with ALS-related declines. Similarly, beta-band increases observed in ALS patients may partly reflect age-related changes in cortical synchronization, rather than disease-specific impairments.

Age variability across study groups can also affect comparisons of spectral-power changes in longitudinal studies, where progressive neural decline in ALS may coincide with normal aging processes. For example, in [44], longitudinal decreases in alpha and theta power might be partly attributable to age, rather than ALS progression, especially if younger patients were not analyzed separately.

To address these challenges, future EEG studies in ALS should ensure rigorous age-matching between ALS patients and controls. Researchers should also use statistical adjustments for age as a covariate in spectral-power analyses, ensuring that results account for the natural effects of aging on brain oscillations. Additionally, stratified analyses based on age groups (e.g., under 50, 50–65, and over 65) could clarify the relative contributions of ALS pathology versus age-related neural changes. Standardizing the age range in participant recruitment will further enhance the comparability and validity of findings across studies.

### 7.3. Influence of Medications

The influence of medications on EEG findings in ALS studies is a critical, yet often overlooked, methodological limitation. ALS patients are frequently prescribed medications to manage symptoms such as spasticity, pain, depression, anxiety, and respiratory issues [81]. These drugs can significantly alter brain oscillatory activity, potentially confounding the interpretation of EEG spectral-power changes as disease-specific.

For instance, drugs such as baclofen (a muscle relaxant used to reduce spasticity) and benzodiazepines (used to manage anxiety) enhance inhibitory GABAergic activity in the brain [82,83]. This can lead to increases in delta and theta power, while reducing or increasing beta activity [84,85], potentially masking or exaggerating ALS-related EEG changes. Similarly, antidepressants (e.g., SSRIs or SNRIs) and anticonvulsants (e.g., gabapentin) influence cortical excitability and connectivity, often altering alpha- and increasing delta- and theta-band oscillations [86,87].

Riluzole, the most commonly prescribed disease-modifying treatment for ALS [88], may also influence EEG patterns. It modulates glutamatergic activity [88], reducing cortical excitability [88], which manifests as changes in alpha power [89]. This may occur by mitigating glutamate excitotoxicity, a key pathological process in ALS. However, these effects remain poorly characterized in EEG research.

Few studies explicitly control for medication effects when analyzing EEG results. While studies [35,40] reported spectral-power differences in ALS patients, they lack detailed information on participants’ medication use, which could confound the observed results. In contrast, study [39] partially accounted for clinical variables, but provided limited data on specific drugs and dosages. This lack of transparency hinders the ability to distinguish medication-related EEG alterations from disease-related changes.

To address this limitation, future studies should systematically record detailed medication data, including type, dosage, and duration of use. Medication effects should be accounted for as covariates in statistical models, to isolate ALS-specific EEG changes. For example, regression models can assess relationships between drug use and changes in specific frequency bands. Researchers should also conduct subgroup analyses to compare EEG results between patients on and off medications (e.g., riluzole or benzodiazepines) to identify any significant differences. Where feasible, monitoring acute medication effects by recording EEG data before and after administering medications could help determine their immediate impact on spectral power.

By systematically accounting for medication effects, researchers can improve the reliability and interpretability of EEG findings in ALS. Additionally, understanding how medications influence EEG patterns may offer new insights into their mechanisms of action and potential neuroprotective effects in ALS.

### 7.4. Lack of Functional- and Behavioral-Outcome Measures

A major methodological limitation in EEG studies of ALS is the insufficient incorporation of comprehensive functional- and behavioral-outcome measures to correlate with EEG findings. While spectral-power alterations in EEG frequency bands (e.g., alpha, beta, and theta) have been consistently reported, their clinical relevance often remains unclear because functional impairments, motor deficits, and behavioral symptoms are not adequately measured or integrated into the analyses.

Functional measures such as the ALS Functional Rating Scale—Revised (ALSFRS-R), Manual Muscle Testing (MRC scores), and Forced Vital Capacity (FVC) are critical for quantifying motor decline—the hallmark of ALS. However, not all studies incorporate these measures. For example, in study [38], EEG spectral-power changes were reported without direct correlation to motor function or disease-severity scores. As a result, while alterations in beta and gamma activity were observed, their relationship to ALS progression remained speculative.

Behavioral and cognitive outcomes, including executive function, verbal fluency, and behavioral impairment (e.g., measured via tools like ECAS or BBI), are also often overlooked in EEG analyses. For instance, the longitudinal study [44] identified distinct EEG changes (e.g., reduced beta synchrony and increased gamma power) in ALS patients with cognitive impairment, linking these findings to functional decline. However, many other studies fail to incorporate detailed cognitive or behavioral evaluations, limiting insights into the impact of EEG changes on non-motor symptoms. This gap is particularly critical, because EEG findings in ALS extend beyond motor areas; abnormalities are frequently observed in frontotemporal and parietal regions, especially in patients with cognitive and behavioral impairments.

Furthermore, studies that focus on specific disease states, such as transitions to the locked-in state (LIS) or completely locked-in state (CLIS), often lack functional correlates. For example, studies [48,49] reported severe EEG slowing (dominant delta and theta activity) and the loss of alpha rhythms in CLIS patients, but they did not quantify residual motor or cognitive function. Without functional measures, it is challenging to interpret these EEG changes as markers of disease progression or assess their clinical significance.

To address these limitations, future EEG studies in ALS should systematically include standardized clinical assessments, such as ALSFRS-R, MRC scores, and FVC, to correlate functional impairments with EEG findings. Integrating cognitive and behavioral assessments (e.g., ECAS, MoCA, or BBI) will help link EEG spectral changes in frontotemporal and parietal regions to cognitive and behavioral symptoms. Longitudinal measurements of both EEG power and functional outcomes are essential to track disease progression and identify EEG markers predictive of clinical decline. Additionally, analyzing subgroup differences, e.g., between patients with cognitive or behavioral impairment and those without, could provide a better understanding of the relationship between EEG abnormalities and ALS phenotypes. By combining EEG spectral analyses with comprehensive clinical, functional, and behavioral outcomes, researchers can establish clearer relationships between neural oscillatory changes and the multifaceted symptoms of ALS. This will not only enhance the clinical relevance of EEG findings, but also improve their potential utility as biomarkers for disease progression and therapeutic monitoring.

### 7.5. Too Few Longitudinal Studies

A critical limitation in EEG studies of ALS is the lack of longitudinal research examining changes in EEG power over time. Most studies employ cross-sectional designs, providing only a single snapshot of cortical oscillatory activity and failing to capture how EEG power evolves with disease progression. This omission limits the ability to identify reliable EEG biomarkers of disease progression or to determine whether specific spectral changes precede functional or cognitive decline.

Longitudinal studies are particularly important in ALS, as the disease manifests with progressive neurodegeneration that involves both motor and non-motor regions of the brain. For example, reductions in alpha and beta power, alongside increases in delta and theta activity, are observed in advanced ALS stages. However, without longitudinal data, it is unclear whether these spectral shifts develop gradually or emerge only in later stages. Study [44] provides some evidence for longitudinal changes, demonstrating decreases in theta power and increases in gamma power in frontal and temporal regions. These findings also showed subgroup-specific patterns, with distinct changes in patients with cognitive impairment, behavioral impairment, or pure motor symptoms. Nevertheless, such studies remain rare, and most findings are limited to a single time point, reducing their generalizability.

Furthermore, there is a lack of studies examining EEG changes during the critical transitions from normal function to LIS and, ultimately, CLIS. These transitions represent extreme endpoints of ALS, where motor abilities are almost entirely lost, but detectable brain activity persists. Studies [48,49] have reported severe slowing of EEG frequencies (increased delta and theta power, absence of alpha rhythms) in CLIS patients. However, these findings were based on very small sample sizes, and lacked longitudinal follow-up, making it difficult to determine when and how EEG changes emerged during the transition. Longitudinal monitoring of EEG power during these critical stages could provide valuable insights into the neurophysiological mechanisms underlying the progression to LIS and CLIS, as well as identify early biomarkers of these transitions.

To address this limitation, future studies should prioritize longitudinal designs that monitor EEG spectral changes over time, ideally from early ALS diagnosis through advanced disease stages. Regular EEG recordings, combined with standardized clinical assessments (e.g., ALSFRS-R, cognitive, and behavioral measures), would allow researchers to correlate spectral-power changes with functional decline and identify trends that predict disease milestones. Additionally, monitoring EEG during transitions from normal function to LIS and CLIS is essential to uncover the temporal dynamics of cortical changes and to determine whether EEG power alterations could serve as early indicators of disease progression. By incorporating longitudinal methodologies, future studies can establish a clearer understanding of how brain activity evolves in ALS, improving the utility of EEG as a biomarker for disease monitoring and providing valuable insights into the neurophysiological changes accompanying functional and motor decline in ALS patients.

### 7.6. Differences in the Definition of Frequency Ranges

A significant methodological limitation in EEG studies of ALS is the inconsistent definition of EEG frequency bands across studies. Frequency ranges for delta, theta, alpha, beta, and gamma bands are not standardized, which introduces variability and complicates comparisons of results between studies. Even subtle differences in band definitions can lead to discrepancies in the reported findings, particularly when analyzing power changes in regions affected by ALS pathology.

For example, beta power is defined differently across studies. In study [35], beta spans 13–25 Hz, while studies [40,43] define it more broadly as 13–30 Hz, and other studies divide it into subranges: low beta (13–20 Hz) and high beta (20–30 Hz). This lack of uniformity can obscure patterns in beta-power changes. Increased beta activity reported in motor and frontotemporal regions in some studies might be restricted to a particular sub-range of the beta band. If beta power is not divided into low and high ranges, subtle changes in specific sub-bands may go undetected, reducing the ability to link spectral power with clinical outcomes, such as motor decline.

A similar issue arises in the definition of alpha bands. While many studies define alpha as 8–13 Hz, others adjust the range slightly, such as 8.5–12.5 Hz in study [37]. Studies that distinguish between low alpha (8–10 Hz) and high alpha (11–13 Hz), such as [45], are better equipped to detect specific changes in subranges, which might otherwise be averaged out. This is especially relevant in ALS, where reductions in high alpha power are frequently observed in central and frontotemporal regions, corresponding to motor and cognitive decline. Failing to separate alpha sub-bands can mask these disease-specific findings.

The definition of gamma power is also inconsistent. While some studies divide gamma into low gamma (30–50 Hz) and high gamma (50–100 Hz), others, such as study [38], use a broad range (30–90 Hz). This variability can obscure important findings, as increases in gamma power observed in frontal or parietal regions may occur predominantly within one subrange. Furthermore, gamma activity is particularly susceptible to artifacts such as muscle activity, and improper band definitions can amplify this issue.

To address these inconsistencies, EEG research in ALS should adopt a standardized framework for defining frequency bands. The following ranges are widely accepted and recommended: delta: 1–4 Hz; theta: 4–7 Hz; alpha: 8–13 Hz (with optional separation into low alpha: 8–10 Hz and high alpha: 11–13 Hz); beta: 13–30 Hz (with optional separation into low beta: 13–20 Hz and high beta: 20–30 Hz); and gamma: 30–100 Hz (with low gamma: 30–50 Hz and high gamma: 50–100 Hz). Where relevant, dividing beta and gamma bands into subranges should be encouraged, to detect subtle but clinically relevant changes. Studies should also explicitly state their frequency-band definitions in Section 2, to improve transparency and facilitate replication. Adopting consistent frequency ranges across EEG studies will enhance the comparability of findings, allowing researchers to draw more reliable conclusions about spectral-power changes in ALS and their associations with disease progression and clinical outcomes.

### 7.7. Insufficient Integration of Multimodal Approaches

The inadequate use of complementary neuroimaging or physiological modalities to supplement EEG data is a significant problem in EEG studies of ALS. While EEG provides valuable insights into cortical oscillations and neural connectivity, it does not capture the complete range of brain abnormalities linked to ALS, such as structural atrophy [90], metabolic dysfunction [16], and changes in cerebral blood flow [91]. Without integrating complementary modalities such as MRI, fNIRS, MEG, or neurochemical analyses, the interpretation of EEG changes remains incomplete. This limitation can result in oversimplified conclusions about ALS pathology. For example, EEG research has repeatedly shown changes in beta activity and decreases in alpha power, especially in the motor and frontotemporal networks. These findings could be better contextualized by pairing them with structural imaging techniques such as diffusion tensor imaging (DTI) or MRI. For instance, reductions in EEG alpha power observed in central regions could be mapped to cortical thinning or white matter damage in the motor cortex. Similarly, increased beta power in frontotemporal regions could be cross-validated with structural or functional abnormalities detected using MRI or fMRI, providing a clearer link between EEG changes and neurodegenerative mechanisms.

Functional near-infrared spectroscopy (fNIRS) is another promising modality for integration with EEG. While EEG records electrical activity, fNIRS provides supplementary information about neurovascular coupling by measuring changes in brain oxygenation and hemodynamic responses. Study [37], which combined fNIRS and EEG, showed that ALS patients had reduced hemodynamic responses and decreased EEG spectral power. This multimodal approach advances our understanding of how altered brain activity and vascular function co-occur in ALS, and may help identify early markers of disease progression.

Magnetoencephalography (MEG) offers another complementary modality with superior temporal and spatial resolution compared to EEG. Combining EEG with MEG could allow for a more accurate examination of oscillatory dynamics and connectivity alterations in ALS patients. For example, MEG could verify whether changes in beta synchronization observed in EEG research are limited to motor areas or encompass larger networks. Despite its potential, no studies have yet combined EEG and MEG in ALS research, likely due to financial and technological barriers.

Additionally, integrating EEG with neurochemical assessments, such as magnetic resonance spectroscopy (MRS) or PET could shed light on the relationship between EEG spectral changes and neurotransmitter imbalances. For instance, increased gamma power observed in some ALS studies may reflect altered glutamatergic activity, a known pathological hallmark of ALS. Combining EEG with MRS to quantify glutamate concentrations or PET to measure GABAergic activity could clarify the neurochemical basis of EEG changes.

To address this limitation, future EEG studies in ALS should adopt multimodal approaches to provide a more comprehensive understanding of disease mechanisms. Specifically, combining EEG with MRI or DTI can correlate spectral-power changes with structural atrophy and white matter degeneration. Integrating EEG with fNIRS can enhance insights into neurovascular function, while combining EEG with MRS or PET can elucidate the neurochemical basis of EEG power changes, such as glutamatergic excitotoxicity or GABAergic inhibition. Using simultaneous EEG and MEG can improve the spatial localization of changed oscillatory activity.

By implementing multimodal approaches, researchers can enhance the spatial and functional interpretation of spectral-power changes, validate EEG results, and learn more about the pathophysiological mechanisms underlying ALS progression. This integrated approach will improve the clinical relevance and reliability of EEG as a biomarker for ALS diagnosis, disease monitoring, and therapeutic evaluation.

### 7.8. Limited Measurement of Gamma Power

One critical limitation in EEG studies of ALS is the inconsistent inclusion and analysis of gamma power (30–100 Hz), which remains underexplored compared to lower-frequency bands such as delta, theta, alpha, and beta. Gamma oscillations are associated with higher-order cognitive functions, including attention [92] and memory processing [92], making them particularly relevant for understanding cognitive and behavioral impairments in ALS patients. Moreover, gamma-band activity reflects local neuronal synchronization, and is tightly linked to GABAergic inhibition [93,94], both of which are often disrupted in ALS pathology. The lack of standardized measurement and reporting of gamma power across studies limits its potential as a biomarker for disease progression, cortical dysfunction, and therapeutic targets.

Some studies, such as [38,40], included gamma power analyses and identified notable alterations. For instance, study [38] reported enhanced high gamma power (50–90 Hz) across cortical regions, particularly in ALS patients with less severe disease stages, suggesting possible compensatory mechanisms. Similarly, study [40] identified increased gamma activity over frontoparietal regions, potentially reflecting maladaptive neural reorganization in response to motor network degeneration. However, other studies either failed to analyze gamma power or omitted it entirely from their results. This exclusion is often due to technical challenges, including the difficulty of distinguishing true gamma oscillations from muscle artifacts (electromyographic contamination), which are prevalent in ALS patients, due to involuntary muscular activity. Without robust artifact removal methods, gamma-power measurements may be confounded, leading to false-positive findings.

Additionally, gamma power is often reported in broad-frequency ranges, such as 30–90 Hz, without distinguishing between low gamma (30–50 Hz) and high gamma (50–100 Hz). This lack of granularity can obscure specific frequency-dependent changes that could hold great clinical relevance. For example, low gamma power is primarily associated with motor network activation, whereas high gamma is linked to cognitive processes and cortical excitability. Dividing gamma power into subranges would enable researchers to detect subtle but meaningful changes and establish connections to ALS phenotypes, such as pure motor ALS or ALS with cognitive or behavioral impairments.

To address this limitation, future studies should prioritize the inclusion of gamma-power analyses in EEG research and implement robust methods to ensure accurate measurements. Specifically, researchers should employ advanced artifact removal techniques, such as Independent Component Analysis (ICA) or machine-learning algorithms, to separate true gamma activity from muscle artifacts. Gamma power should be analyzed in both low-gamma (30–50 Hz) and high-gamma (50–100 Hz) subranges, to identify frequency-specific alterations and their association with motor or cognitive impairments. Comparative analyses across ALS subgroups, such as spinal-onset, bulbar-onset, and ALS-FTD (ALS with frontotemporal dementia), can reveal whether gamma-power alterations are linked to specific phenotypes. Longitudinal studies are needed to track changes in gamma activity over time and assess its role in disease progression, compensatory mechanisms, and cortical reorganization. Furthermore, combining gamma-power analysis with multimodal approaches (e.g., MRS for glutamate measurements or fNIRS for cortical oxygenation) could provide insights into the neurochemical and metabolic processes underlying gamma oscillations in ALS. By including gamma-power analyses more consistently and applying rigorous methodological frameworks, future research can determine the role of gamma oscillations as a biomarker for ALS.

### 7.9. Influence of Comorbidities

Another major limitation in EEG studies of ALS is the frequent omission of comorbidities, such as anxiety disorders, depression, sleep disturbances, chronic pain, and cognitive impairment, which are common in ALS patients and can significantly influence EEG power spectra. These conditions often coexist with ALS, contributing to alterations in cortical activity that may be misattributed to the disease itself. Failure to measure, report, and account for comorbidities introduces confounding factors, complicating the interpretation of EEG findings and reducing their reliability as biomarkers of ALS progression.

For example, depression and anxiety—common psychological responses to ALS [95,96]—are known to alter brain oscillatory activity. Depression often correlates with reduced alpha power [97], whereas anxiety is linked to heightened beta power [98]. Without accounting for these comorbidities, EEG studies may incorrectly attribute observed changes in alpha or beta power to ALS-related pathology rather than underlying mood disorders.

Sleep disturbances, such as insomnia or sleep apnea, are also prevalent in ALS [99] and can significantly affect EEG power, particularly an increase in delta power due to sleep deprivation [100]. Given that many EEG recordings are performed at rest, underlying sleep disruptions may affect baseline cortical oscillations, further confounding results.

Additionally, chronic pain—a frequently under-reported symptom in ALS [101,102]—can alter cortical activity, increasing theta and alpha power in resting-state EEG studies [103]. If pain levels are not assessed, changes in high-frequency oscillations such as beta and gamma power might reflect the influence of chronic pain rather than ALS-specific cortical dysfunction.

To address these issues, future EEG studies in ALS must systematically measure and control for comorbidities during data collection and analysis. Researchers should implement standardized clinical assessments for depression (e.g., the Beck Depression Inventory), anxiety (e.g., the Generalized Anxiety Disorder Scale), and pain levels (e.g., the Numeric Pain Rating Scale). They should also use validated tools to assess sleep disturbances (e.g., the Pittsburgh Sleep Quality Index) and consider excluding patients with severe sleep disorders or accounting for sleep quality in statistical models. These comorbidities should then be included as covariates in EEG analyses, to disentangle their effects from ALS-related spectral changes. By systematically identifying and controlling for comorbidities, future research will improve the specificity of EEG findings, ensuring that changes in cortical oscillations are more accurately attributed to ALS pathology. This approach will strengthen the reliability of EEG as a diagnostic and monitoring tool, while providing a clearer understanding of the neurophysiological mechanisms underlying ALS progression.

### 7.10. Small Patient Samples

One of the most significant limitations in EEG studies of ALS is the reliance on small patient samples, which restricts the generalizability, statistical power, and robustness of the findings. Given the heterogeneity of ALS in terms of disease onset, progression rate, motor dysfunction, and cognitive or behavioral impairments, small sample sizes often fail to capture the full spectrum of EEG alterations associated with ALS pathology. As a result, studies may overestimate or underestimate the significance of specific EEG changes, limiting their potential as biomarkers of disease progression, severity, or phenotype differentiation.

In many studies, sample sizes ranged from 6 to 20 patients, as seen in studies [37,38,42,45]. While these investigations provide valuable preliminary insights, such small cohorts are insufficient to detect subtle but clinically relevant changes in EEG power spectra across ALS subgroups. For example, differences in alpha power between ALS patients and controls may fail to achieve statistical significance simply due to insufficient statistical power to detect small effect sizes. Similarly, studies examining beta or gamma-power alterations, such as [39,40], report trends without robust conclusions, as limited sample sizes introduce high variability in EEG measurements.

The impact of small sample sizes is further compounded by ALS’s clinical and genetic heterogeneity. Patients with spinal-onset, bulbar-onset, and ALS-FTD may exhibit distinct EEG patterns that cannot be adequately explored in small cohorts. For instance, changes in gamma power observed in ALS-FTD patients, as noted in studies [40,44], might differ from those with purely motor impairments. Without larger, subgroup-specific samples, it becomes challenging to link spectral EEG changes to particular clinical or genetic phenotypes. Additionally, small sample sizes are particularly problematic for longitudinal studies, where patient attrition over time can further reduce the cohort size, limiting the ability to track EEG changes across disease progression or during the transition to LIS or CLIS.

To address these limitations, future studies must prioritize multicenter collaborations and standardized EEG protocols to facilitate data pooling across research sites. Collaborative efforts can enable the creation of larger, more diverse patient cohorts, improving statistical power and enhancing the generalizability of EEG findings. These collaborations should aim to include ALS subgroups (e.g., spinal-onset, bulbar-onset, and ALS-FTD) to ensure that EEG variability reflects true pathological differences, rather than sample limitations. Furthermore, meta-analyses of existing EEG studies can help synthesize results from smaller studies, identifying consistent patterns in EEG power changes across ALS populations.

## 8. Conclusions

The field of resting-state EEG research in ALS is advancing, as evidenced by the inclusion of 17 studies in this mechanistic review. While the included studies differed in methodology, ALS progression stage, and patient characteristics, a consistent biomarker of ALS in resting-state EEG was found to be decreased alpha power. In other frequency bands, findings are less consistent and sometimes contradictory.

Three mechanisms have been proposed to explain the reduction in alpha power in ALS pathophysiology. The most important is increased cortical excitability, a well-documented phenomenon in this condition. Despite the progress made, further confirmation and solidification of these findings require addressing the methodological limitations identified in this review, including small sample sizes, inconsistent frequency-band definitions, and insufficient functional outcome measures. By adhering to the recommendations outlined in this review, future studies can better characterize EEG patterns in ALS, enhance the reliability of EEG biomarkers, and contribute to a deeper understanding of this severe and incurable disorder.

## Figures and Tables

**Table 1:** Studies included in the review.

Ref.	Participants (ALS vs. Controls)	ALS Subtype(s)	EEG Condition (Eyes)	Frequency Bands	Key Clinical Measures	Main Findings
[35]	15 ALS (mean age 67.9 ± 10.9) vs. 15 HC (mean age 68.4 ± 8.0)	Not specified	Eyes closed	δ (1–4 Hz), θ (4–8 Hz), Low-α (IAF–2 Hz to IAF), High-α (IAF to IAF+2 Hz), β (13–25 Hz)	ALSFRS-R, MRC, FVC, ALSAQ-5, King’s, MiToS, MoCA	Higher β-power correlated with worse clinical scores, greater disease burden, and disease progression. No differences in IAF or other bands between ALS and HC.
[36]	95 ALS (21 bulbar, 4 respiratory, 70 spinal) vs. 77 HC	Bulbar, respiratory, spinal-onset	Eyes open	δ (2–4 Hz), θ (5–7 Hz), α (8–13 Hz), β (14–30 Hz), γ (γl:31–47 Hz, γh:53–97 Hz)	ALSFRS-R, ECAS, BBI	Four ALS subphenotypes with distinct EEG signatures. Increased β-power in frontotemporal areas, altered α/γ-band synchrony. Clusters with more network disruption had worse clinical outcomes and shorter survival.
[37]	10 ALS (mean age 58.2 ± 11.6) vs. 9 HC (mean age 61.0 ± 3.8)	Not specified	Eyes closed	δ (0.5–3.5 Hz), θ (3.5–8.5 Hz), α (8.5–12.5 Hz), β (12.5–30 Hz)	Not specified	Significant reduction in θ and α power in ALS, especially in frontal, temporal, parietal, and occipital regions.
[38]	6 ALS (mean age 64) vs. 37 HC	Not specified	Eyes open	δ (1–4 Hz), θ (4–8 Hz), α (8–14 Hz), β (20–30 Hz), γlow (30–50 Hz), γhigh (50–90 Hz)	ALSFRS-R	ALS patients showed increased high γ-power and lower α-power, with spatial differences noted across cortical areas. One patient with severe progression had reduced γ-power.
[39]	18 ALS (mean age 65.75) vs. 38 HC (mean age 66)	Not specified	Eyes open	β (14–30 Hz)	ALSFRS-R, LMN/UMN scores, FMF	Reduced β-power in ALS sensorimotor network. Higher β-power associated with greater LMN impairment and faster disease progression. No correlation with UMN impairment.
[40]	74 ALS (56 spinal, 15 bulbar, 3 respiratory) vs. 47 HC (mean age ~58)	Spinal, bulbar, respiratory	Eyes open	δ (2–4 Hz), θ (5–7 Hz), α (8–13 Hz), β (14–30 Hz), γ (γl:31–47 Hz, γh:53–97 Hz)	Not specified	Reduced spectral power (δ to β-bands) in ALS, notable in occipital, temporal, orbitofrontal, and sensorimotor regions. EEG measures could distinguish ALS subtypes from HC.
[41]	100 ALS (78 spinal, 15 bulbar, 7 ALS-FTD) vs. 34 HC	Spinal, bulbar, ALS-FTD	Eyes open	δ (2–4 Hz), θ (5–7 Hz), αl (8–10 Hz), αh (11–13 Hz), βl (14–20 Hz), βh (21–30 Hz), γ (γl:31–47 Hz, γh:53–97 Hz)	Not specified	Reduced θ-power over bilateral motor areas, increased γ-power in frontoparietal regions. Differences noted across ALS phenotypes.
[42]	21 ALS (mean age 66) vs. 16 HC (mean age 65)	Not specified	Eyes closed	δ (1–4 Hz), θ (4–8 Hz), α (8–13 Hz), β (13–30 Hz)	Not specified	No significant differences in power spectra between ALS and HC in any band.
[43]	18 ALS (15 spinal, 2 respiratory, 1 bulbar; mean age 56) vs. 17 HC (mean age 51)	Spinal, respiratory, bulbar	Eyes open	δ (1–3 Hz), θ (4–7 Hz), α (8–13 Hz), low β (14–21 Hz), high β (22–30 Hz), γ (31–60 Hz)	Not specified	ALS patients had increased α-power (parietal), increased θ-power (frontal/central), and elevated γ-power (frontal, parietal, occipital) compared to HC.
[44]	124 ALS (ALSncbi = 53, ALSci = 27, ALSbi = 58)	Cognitive and behavioral subtypes	Eyes open	δ (2–4 Hz), θ (4–7 Hz), α (7–13 Hz), β (13–30 Hz), γl (30–47 Hz), γh (53–97 Hz)	Fine motor symptoms, cognitive/behavioral assessments	Decrease in θ-power over time (temporal), increase in γ-power (frontal/temporal). ALSbi showed increased γ-power in the temporal lobe. Changes correlated with fine motor symptom progression.
[45]	10 ALS in CLIS (mean age 47.1) vs. 7 HC (mean age 45.7)	CLIS	Eyes closed	δ (1–3 Hz), θ (4–7 Hz), low α (8–10 Hz), high α (11–13 Hz), β (14–30 Hz), γ (31–40 Hz)	Not specified	Significant reduction in high α, β, γ bands in CLIS patients, with dominance of slower oscillations (δ and θ). Trend towards more slow-dominated EEG with longer disease duration.
[46]	18 ALS (mean age 61.7) vs. 14 HC (mean age 63.4)	Not specified	Not specified	δ (1.5–4.0 Hz), θ (4.5–7.5 Hz), α (8.0–13.0 Hz), β (13.5–30 Hz)	Not specified	No global difference in power. Regionally reduced α-power in central areas for ALS. No differences in δ, θ, β.
[47]	12 ALS (mean age 46.75) vs. 9 HC (mean age 47.17)	Not specified	Eyes closed	α (8–13 Hz)	Not specified	ALS patients showed 60–70% lower α-band power than HC, more pronounced in the left hemisphere.
[48]	3 ALS patients (2 in CLIS, 1 LIS→CLIS transition; ages 24–40)	CLIS, LIS→CLIS	Eyes closed	δ (0–4 Hz), θ (4–8 Hz), α (8–12 Hz), low β (12–20 Hz), high β (20–30 Hz), γ (30–45 Hz)	Not specified	CLIS patients showed EEG shifted towards δ/θ. The LIS→CLIS patient maintained α activity initially, but reduced signal strength as disease progressed. Significant spectral differences between CLIS and LIS→CLIS states.
[49]	4 ALS-CLIS patients (ages 29–75)	CLIS	Eyes open and closed	Not specified	Not specified	Pronounced slowing of frequencies, reduced/absent α-waves, synchronized slow waves (~4 Hz) dominating EEG.
[50]	10 ALS (2 CLIS, 8 non-CLIS; mean age 51.5) vs. 10 HC (mean age 61.4)	CLIS, non-CLIS	Eyes open and closed	Not specified	Not specified	CLIS patients showed significant APF slowing (7 Hz and 3.8 Hz), reduced typical α-range activity, and dominance of θ and low α frequencies. Differed significantly from HC and non-CLIS ALS.
[51]	13 LIS (mean age 51) vs. 15 HC (mean age 50.3)	LIS	Eyes closed	δ (IAF-8 to IAF-6 Hz), θ (IAF-6 to IAF-4 Hz), α1 (IAF-4 to IAF-2 Hz), α2 (IAF-2 to IAF Hz), α3 (IAF to IAF+2 Hz), β1 (13–20 Hz), β2 (20–30 Hz)	Not specified	LIS patients had reduced α2 and α3 power and increased δ power compared to HC, indicating slowing of EEG oscillations and reduced higher-frequency activity.

Abbreviations: ALS: Amyotrophic Lateral Sclerosis; HC: Healthy Controls; CLIS: Completely Locked-In State; LIS: Locked-In State; ALSFRS-R: ALS Functional Rating Scale—Revised; LMN/UMN: Lower/Upper Motor Neuron; FMF: Fine Motor Function; ECAS: Edinburgh Cognitive and Behavioural ALS Screen; BBI: Beaumont Behavioural Inventory; IAF: Individual Alpha Frequency; APF: Alpha Peak Frequency.

## Data Availability

No new data were created or analyzed in this study. Data sharing is not applicable to this article.
